# Association between preoperative anxiety and postoperative delirium in older patients: a systematic review and meta-analysis

**DOI:** 10.1186/s12877-023-03923-0

**Published:** 2023-03-30

**Authors:** Ke-Lu Yang, Elke Detroyer, Bastiaan Van Grootven, Krizia Tuand, Dan-Ni Zhao, Steffen Rex, Koen Milisen

**Affiliations:** 1 Department of Public Health and Primary Care, Academic Centre for Nursing and Midwifery, KU Leuven - University of Leuven, Kapucijnenvoer 35/4, B-3000 Leuven, Belgium; 2 Department of Geriatric Medicine, University Hospitals Leuven, Leuven, Belgium; 3 KU Leuven Libraries - 2Bergen - Learning Centre Désiré Collen, Leuven, Belgium; 4 The Second Clinic School, Lanzhou University, Lanzhou, China; 5 Department of Anesthesiology, University Hospitals of Leuven, Leuven, Belgium; 6 Department of Cardiovascular Sciences, KU Leuven - University of Leuven, Leuven, Belgium

**Keywords:** Older patients, Postoperative delirium, Preoperative anxiety, Meta-analysis, Cognitive dysfunction

## Abstract

**Background:**

Postoperative delirium (POD) is a common postoperative complication associated with multiple adverse consequences on patient outcomes and higher medical expenses. Preoperative anxiety has been suggested as a possible precipitating factor for the development of POD. As such, we aimed to explore the association between preoperative anxiety and POD in older surgical patients.

**Methods:**

Electronic databases including MEDLINE (via PubMed), EMBASE (via Embase.com), Web of Science Core Collection, Cumulative Index to Nursing and Allied Health Literature (CINAHL Complete; via EBSCOhost) and clinical trial registries were systematically searched to identify prospective studies examining preoperative anxiety as a risk factor for POD in older surgical patients. We used Joanna Briggs Institute Critical Appraisal Checklist for Cohort Studies to assess the quality of included studies. The association between preoperative anxiety and POD was summarized with odds ratios (ORs) and 95% confidence intervals (CIs) using DerSimonian-Laird random-effects meta-analysis.

**Results:**

Eleven studies were included (1691 participants; mean age ranging between 63.1–82.3 years). Five studies used a theoretical definition for preoperative anxiety, with the Anxiety subscale of Hospital Anxiety and Depression Scale (HADS-A) as the instrument being most often used. When using dichotomized measures and within the HADS-A subgroup analysis, preoperative anxiety was significantly associated with POD (OR = 2.17, 95%CI: 1.01–4.68, I^2^ = 54%, Tau^2^ = 0.4, *n* = 5; OR = 3.23, 95%CI: 1.70–6.13, I^2^ = 0, Tau^2^ = 0, *n* = 4; respectively). No association was observed when using continuous measurements (OR = 0.99, 95%CI: 0.93–1.05, I^2^ = 0, Tau^2^ = 0, *n* = 4), nor in the subgroup analysis of STAI-6 (six-item version of state scale of Spielberger State-Trait Anxiety Inventory, OR = 1.07, 95%CI: 0.93–1.24, I^2^ = 0, Tau^2^ = 0, *n* = 2). We found the overall quality of included studies to be moderate to good.

**Conclusions:**

An unclear association between preoperative anxiety and POD in older surgical patients was found in our study. Given the ambiguity in conceptualization and measurement instruments used for preoperative anxiety, more research is warranted in which a greater emphasis should be placed on how preoperative anxiety is operationalized and measured.

**Supplementary Information:**

The online version contains supplementary material available at 10.1186/s12877-023-03923-0.

## Introduction

Postoperative delirium (POD) is an adverse complication that manifests as acute and fluctuating alterations of mental status, involving disturbances in attention, consciousness*,* and cognition [1–3]. The incidence of POD varies widely depending on the population and surgical procedure under study. Among older patients undergoing cardiac and major noncardiac surgery, the incidence of POD is reported to be as high as 60% [1, 4]. POD is associated with multiple adverse outcomes, including an excess in morbidity and mortality, an increase in the duration of hospitalization, and higher rates of readmission and functional decline, as well as increased levels of dependency in activities of daily living post-discharge [5–7]. Healthcare costs caused by or associated with delirium are estimated to equal more than 182 billion Euros per year in Europe and up to 164 billion dollars per year in the USA [8].

As a consequence, the prevention of POD is essential. Risk factors for POD can be divided into two parts; predisposing factors and precipitating factors. Predisposing factors are risk factors intrinsically related to the patient, and usually exist already prior to admission. Precipitating factors are risk factors that trigger the onset of delirium after admission, comprising non-surgical and surgical factors [2, 9]. The latter factors are principally amenable for prevention strategies. Preoperative anxiety, suggested to be a possible precipitating factor, is usually defined by “state anxiety symptoms” reflecting a temporal and transient emotional state that varies in intensity in response to environmental stimuli [10–13]. Notably, when measuring anxiety in the face of a specific event (e.g. a surgical procedure), it is important to distinguish this type of anxiety with trait anxiety, which can be defined as an anxious personality [14]. It is reported that 48 to 56% of patients admitted for surgery experience preoperative anxiety, and even reaches 85% in day surgery patients, which may cause hemodynamic change during anesthetic induction as well as increase the requirement of anesthetics introperatively, leading to an increased risk of postoperative complications [15–21]. Given the high prevalence of preoperative anxiety, abundant protocols to reduce anxiety have been administrated before surgery including pharmacological therapy and non-pharmacological therapy. Unfortunately, anxiolytic premedication, especially benzodiazepines, has been proven to be significantly associated with POD despite being a common way of anxiety reduction [22, 23]. Non-pharmacological therapy has shown its therapeutic potential for preoperative anxiety and safety compared to pharmacologic therapy, such as cognitive-behavioral therapy, music therapy, preoperative patient education, massage, etc [24, 25]. However, whether the anxiety reduction of these kind of preoperative interventions also might reduce the risk of POD and more specifically, if there is a true relationship between preoperative anxiety and POD still needs to be elaborated. Currently, the pathophysiology of the relationship between preoperative anxiety and POD remains elusive. Studies suggested that the migration of peripheral inflammatory cytokines into the central nervous system and the interaction of cytokines with microglia may induce neuroinflammation and subsequent delirium, which has also been implicated in anxiety [10, 26–28]. Besides, anxiety may also be related to higher glucocorticoid concentration, and metabolic derangements are well-known mechanisms contributing to delirium [28]. Only a few studies have explored this relationship but failed to show a conclusive association between preoperative anxiety and POD due to methodological problems, such as small sample sizes and inappropriate tools for the assessment of either POD or anxiety [12, 29]. In contrast, more recent studies have observed a link between preoperative anxiety and POD in older surgical patients, reporting large effect sizes despite small sample sizes [10, 11]. The various assessment tools and conceptual issues with regard to the evaluation of preoperative anxiety and POD may explain the heterogeneity of findings, such as inconsistent operational definitions of preoperative anxiety and unclear time periods for measuring preoperative anxiety.

Therefore, the hypothesis of an association between preoperative anxiety and POD in older surgical patients awaits rigorous testing. To resolve this controversy, we performed a systematic review and meta-analysis of prospective studies in older surgical patients aiming to elucidate the role of preoperative anxiety in the development of POD.

## Methods

The protocol has been registered in the PROSPERO database (CRD42020198068) [30]. This manuscript was written in accordance with the Preferred Reporting Items for Systematic reviews and Meta-Analyses (PRISMA) 2020 reporting statement [31].

### Search strategy

The databases MEDLINE (via PubMed), EMBASE (via Embase.com), Web of Science Core Collection, Cumulative Index to Nursing and Allied Health Literature (CINAHL Complete; via EBSCOhost), were searched from inception to 25th of July 2022, using specified terms for anxiety AND delirium AND (preoperative OR postoperative OR perioperative OR surgery) (the search strategies for each database were drawn up in collaboration with an experienced information specialist from the library and are listed in Additional file 1). We also used control articles [12, 13, 32] to check the rationality of search strategies. There was no restriction on publication date and language. Cochrane Central Register of Controlled Trials (CENTRAL, 2021, Issue 9; via Cochrane Library), the World Health Organization International Clinical Trials Registry Platform (WHO-ICTRP) (https://trialsearch.who.int), and ClinicalTrials.gov (https://clinicaltrials.gov/) were also searched. Additionally, the reference lists of included studies were manually screened.

### Eligibility criteria and study selection

Studies were eligible if they investigated the role of preoperative anxiety as a risk factor for the development of POD among older surgical patients using prospective designs. For inclusion, studies had to fulfill the following criteria: 1) The study population consisted of surgical patients 60 years of age and older, or had an average or median age of at least 60 years; 2) the studies had to use validated assessment instruments for preoperative anxiety and POD, or application of Diagnostic and Statistical Manual of Mental Disorders criteria [3] by a trained professional (e.g., a psychiatrist); 3) The interval between assessment of preoperative anxiety and surgery had to be no longer than seven days; besides, studies that mentioned preoperative anxiety but did not specify the precise time period of assessment prior to surgery were initially considered eligible. In these cases, we contacted the authors to obtain relevant information. Qualitative studies, review articles and conference abstracts were excluded.

Two reviewers and a medical student screened the titles and abstracts of records via “Rayyan” [33] and reviewed the full text of all potential studies independently. Disagreements were solved through discussion within the entire research group.

### Data extraction

Two reviewers independently extracted data from the included studies. The data extraction sheet included the first author, year of publication, study design, study site, mean age, sample size, gender proportion, type of surgery, assessment tools of preoperative anxiety and POD, and details of the assessments. We also extracted odds ratio (OR) with the 95% confidence interval (CI) from the multivariable-adjusted models and univariate analysis. We further accessed original data from studies that did not report OR to reduce a source of heterogeneity.

### Quality assessment of individual study

Two reviewers independently assessed the quality of included studies using the Joanna Briggs Institute (JBI) Critical Appraisal Checklist for Cohort Studies [34]. The checklist encompasses 11 questions regarding the internal validity of study, with the option to answer “yes” (good quality); “no” (poor quality); “unclear”; or “not applicable”. We also added one extra item regarding whether a theoretical definition of preoperative anxiety was used in the study (i.e., a clear sentence aiming to define preoperative anxiety in the study of intrest). All the disagreements were solved by discussion.

### Data synthesis and statistical analysis

A narrative synthesis and descriptive summary tables were used to describe the study characteristics, quality assessment and findings of the studies. Continuous variables were described as mean with standard deviation (SD), and dichotomous variables were reported as the number of cases and percentages. For meta-analysis, we used adjusted ORs and 95%CI to calculate the relationship between preoperative anxiety and POD via the DerSimonian-Laird random-effects meta-analysis with the heterogeneity calculated by inverse variance method. When more than one adjusted OR was reported, the ratio with the highest number of confounders was selected. We performed separate analyses using different measurement levels of preoperative anxiety assessment (i.e. use of continuous scores versus use of proportions of anxious patients with a score above a specified cut off point), as well as subgroup analyses based on different anxiety assessment tools. We used forest plots to display the results of meta-analysis via Stata version 14.0 (StataCorp, College Station, Texas). Statistical heterogeneity was examined with Cochrane’s Q test and I^2^ value with assigned adjectives of low, moderate, and high to 25%, 50%, and 75% of I^2^ value, respectively [35], and Tau^2^ with a value of 0 indicating no between study variance (i.e. no hererogeneity). We also conducted a sensitivity analysis by excluding the study that failed to report the assessment time period of preoperative anxiety. Publication bias was not assessed because of the number of included studies in all meta-analyses was less than 10 studies, which may underpower the test [36]. Considering that measurement levels were inconsistent between the included studies, we reanalyzed the data from Detroyer 2008 [12] and Milisen 2020 [13], previously published by our research team, in terms of better homogeneity by conducting a multivariate logistic regression analysis adjusting for the same confounders as in the original analysis [12, 13]. We calculated the 6-item version of STAI instead of full version of STAI-S for Detroyer 2008 [12] so that it could correspond with the data from Van Grootven 2016 [32], and we also recalculated the adjusted OR of dichotomized measurement of HADS-A for Detroyer 2008 [12] and dichotomized measurement of APAIS-A for Milisen 2020 [13].

## Results

### Study selection and characteristics

**Fig. 1:**
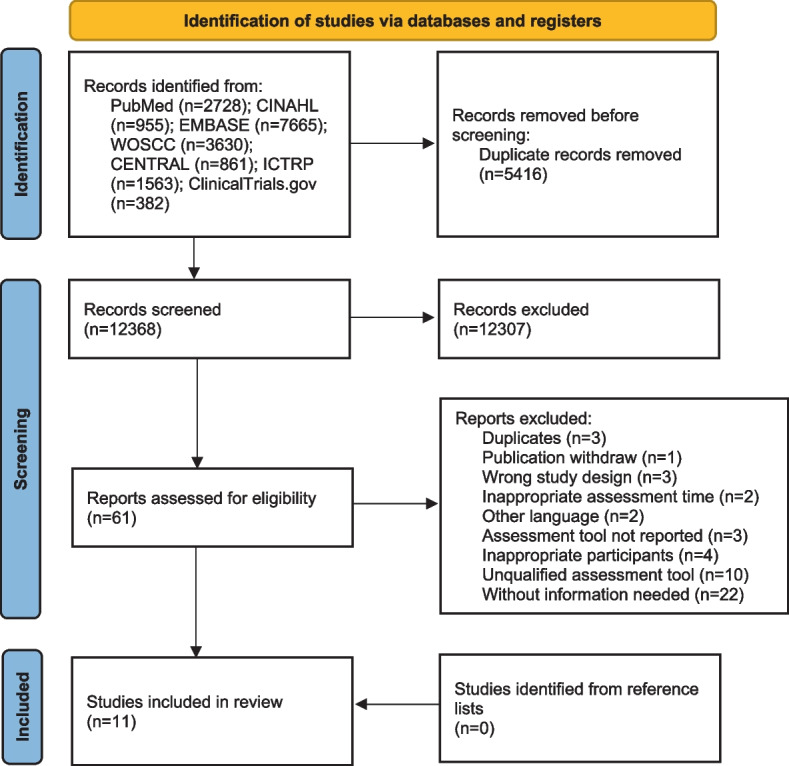
Flow diagram of study selection. CINAHL, Cumulative Index to Nursing and Allied Health Literature; WOSCC, Web of Science Core Collection; CENTRAL, Cochrane Central Register of Controlled Trials; ICTRP, the World Health Organization International Clinical Trials Registry Platform

We identified 15,839 potential records from databases. After removing duplication and excluding ineligible studies according to the inclusion and exclusion criteria, a total of 10 studies could be included [10–13, 29, 32, 37–40]. Because of the low number of studies we could include, we added an additional 11th study [41] that fulfilled all criteria except for one, i.e. preoperative anxiety being measured on 47.4 ± 30.5 days (mean ± standard deviation) before surgery instead of one week before surgery as required in our inclusion criteria. Figure 1 shows the process of study selection and the reasons for exclusion of records during the full-text screening. In addition, a total of 1945 potential records could be identified from study registry websites, but no records matched the criteria.

These 11 studies enrolled in total 1691 participants with a mean age between 63.1–82.3 years undergoing cardiac surgery (*n* = 6) [12, 13, 37, 38, 40, 41], hip fracture surgery (*n* = 2) [29, 32], orthopedic surgery (*n* = 1) [11], tumor resection surgery (*n* = 1) [10], and prolapse surgery (*n* = 1) [39]. Nine prospective cohort studies [10–12, 29, 37–41] and two secondary data analyses of prospective studies [13, 32] were conducted in Belgium (*n* = 3) [12, 13, 32], the Netherlands (*n* = 2) [29, 37], China (*n* = 2) [11, 38], Japan (*n* = 2) [10, 41], United States of America (*n* = 1) [39], and Sweden (*n* = 1) [40]. More details can be found in Table 1.

**Table 1:** Characteristics of included studies

Author year/ location	Participants				Assessment tools		Assessment time		Assessment results	
	Age (mean ± SD), y	Sample size (ND/D), n	Sex, Male, n (%)	Type of surgery	Preop-erative anxiety	Postop-erative delirium	Preoperative anxiety	Postoperative delirium	Preoperative anxiety	Postopera-tive delirium
Bakker 2012 [37]/ Netherlands^a^	76.2 ± 3.8	201 (138/63)	121 (60)	cardiac surgery^c^	HADS-A	CAM-ICU	the day before surgery	day 2–7 postoperat-ively or discharge	score = 6.9 ± 2.8 for ND/7.0 ± 2.3 for D^e^	IR = 31%, duration = 3.3 days^f^
Cheng 2021 [38]/ China^a^	63.1 ± 11.2	152 (142/10)	104 (68)	cardiac surgery^c^	HADS-A	CAM-ICU	the day before surgery	day 1–5 postoperatively or until transferred out of the ICU	IR = 19%	IR = 7%, duration = 3 days^e^
Detroyer 2008 [12]/ Belgium^a^	71 (8)^f^	104 (77/27)	82 (79)	cardiac surgery^c^	STAI-S	CAM; CAM-ICU	the day before surgery	day 1, 3, and 7 postoperatively	IR = 56%, score = 39.5 (16)^f^	IR = 26%, duration = 2(4) days^f^
Milisen 2020 [13]/ Belgium^b^	75.7 ± 5.9	190 (112/78)	99 (52)	cardiac surgery^c^	APAIS-A; VAS-A	3D-CAM; CAM-ICU; ICDSC; DOS	the day before surgery	day 1–5 postoperatively	IR = 31% score (APAIS-A) = 9.1 ± 3.8^e^	IR = 41%
Ren 2021 [11]/ China^a^	74.2 ± 7.3	263 (190/73)	74 (28)	orthope-dic surgery^c^	HADS-A	CAM; CAM-ICU; RASS	not mentioned	day 1–5 postoperatively	IR = 15%	IR = 28%, duration = 2 days^f^
Slor 2013 [29]/ Netherlands^a^	82.3 ± 5.7	53 (30/23)	12 (23)	hip fracture surgery^d^	HADS-A	CAM; DRS-R-98	within 12 h after admission, but before surgery	day 1–5 postoperatively or discharge	score = 9.8 ± 2.5 for ND/8.8 ± 1.7 for D^e^	IR = 43%, duration = 2 days^e^
Van Grootven 2016 [32]/ Belgium^b^	80.1 ± 6.8	86 (62/24)	21 (24)	hip fracture surgery^d^	STAI-6	CAM; DI	within 24 h after admission, but before surgery	day 1, 3, 5, and 8 postoperatively	score = 12.3 ± 2.1^e^	IR = 28%, duration = 2(1) days^f^ severity score = 4(3)^f^
Wada 2019 [10]/ Japan^a^	66.0 ± 10.0	91 (62/29)	62 (68)	tumor resecti-on surgery^c^	HADS-A	DSM-V; DRS-R-98; DMSS	the day before surgery	day 1–5 postoperatively	IR = 24%, score = 4.3 ± 3.0 for ND/5.3 ± 3.5 for D^e^	IR = 32%, duration = 2.3 ± 1.8 days^e^, severity score = 8.4 ± 4.2^e^
Ackenbom 2022 [39]/USA^a^	72.5 ± 6.1	165 (145/20)	0	prolapse surgery^c^	BAI	CAM	within two weeks before surgery	day 1–7 postoperatively	Score = 4 (2–8.5)^f^ for ND/6.5 (3–12)^g^ for N	IR = 12%
Segernäs2022 [40]/Sweden^a^	72.1 ± 6.2	218 (171/47)	159 (73)	cardiac surgery^c^	HADS-A	CAM-ICU; Nu-DESC	before surgery	day 1–7 postoperatively	Score = 4 (2–8.5)^g^	IR = 22%
Fukunaga 2022 [41]/Japan^a^	74.9 ± 6.1	168 (142/26)	93 (55)	cardiac surgery^c^	STAI-S	DSM-V	47.4 ± 30.5 days^e^ before surgery	day 1–3 postoperatively	score = 37.0 ± 7.9 for ND/36.6 ± 8.3 for D^e^	IR = 16%

*SD* standard deviation, *IQR* interquartile range, *ND* non-delirium, *D* delirium, *STAI-S* State scale of Spielberger State-Trait Anxiety Inventory, *STAI-6* 6-item version of state scale of STAI; *APAIS-A* Anxiety subscale of Amsterdam Preoperative Anxiety and Information Scale, *VAS-A* Visual Analogue Scale for anxiety, *HADS-A* Anxiety subscale of Hospital Anxiety and Depression Scale, *BAI* Beck Anxiety Inventory, *CAM* Confusion Assessment Method, *CAM-ICU* CAM for the Intensive Care Unit, *3D-CAM* 3-Minute Diagnostic Interview for CAM delirium, *ICDSC* Intensive Care Delirium Screening Checklist, *DI* Delirium Index, *DOS* Delirium Observation Scale, *DSM-V* Diagnostic and Statistical Manual of Mental Disorders criteria fifth edition, *DRS-R-98* Delirium Rating Scale Revised-98, *DMSS* Delirium Motor Subtype Scale, *RASS* Richmond Agitation Sedation Scale, *IR* incidence rate

^a^Cohort

^b^Secondary data analysis of prospective studies

^c^Elective surgery

^d^Emergency surgery

^e^mean ± SD

^f^median (interquartile range)

^g^median (range)

### Quality assessment

As shown in Table 2, the overall quality of included studies was assessed as moderate to good, whereas there were some deficits as well. Four studies [11, 29, 37, 40] were rated as “unclear” in question 3 (Q3) of JBI checklist and Q7 for failing to report whether preoperative anxiety and POD were measured by trained raters. Besides, Five studies [10, 38–41] were “unclear” whether patients with pre-operative delirium were excluded (Q6). In terms of follow-up, Bakker 2012 [37] and Segernäs 2022 [40] didn’t report if there were participants lost during follow-up, and Slor 2013 [29] didn’t explicitly state how long the in-hospital period was (Q9 and Q10). Regarding the questions rated as “not applicable”, no patients were lost to follow-up in Cheng 2021 [38] (Q 10), and the primary objective of Slor 2013 [29] was not focused on the relationship between preoperative risk factors and POD (Q5 and Q11). In the study of Fukunaga 2022 [41] POD was evaluated within two / three days after surgery which was rather short considering POD may occur up to one week postoperatively (Q8). The theoretical definitions of preoperative anxiety were only mentioned in five studies [11–13, 32, 41] as described in Table 2.

**Table 2:** The results of quality assessment

Author year	Q1	Q2	Q3	Q4	Q5	Q6	Q7	Q8	Q9	Q10	Q11	Theoretical definition of preoperative anxiety
Bakker 2012 [37]	Y	Y	U	Y	Y	Y	U	Y	U	U	Y	Not mentioned
Cheng 2021 [38]	Y	Y	Y	Y	Y	U	Y	Y	Y	NA	Y	Not mentioned
Detroyer 2008 [12]	Y	Y	Y	Y	Y	Y	Y	Y	Y	Y	Y	Preoperative state anxiety reflects a temporal and transient emotional state with changing intensity as a reaction to environmental stimuli.
Milisen 2020 [13]	Y	Y	Y	Y	Y	Y	Y	Y	Y	Y	Y	Preoperative anxiety is defined as “state anxiety (e.g., situational anxiety) symptoms, reflecting a temporal and transient emotional state with changing intensity as a reaction to environmental stimuli”.
Ren 2021 [11]	Y	Y	U	Y	Y	Y	U	Y	Y	Y	Y	Preoperative anxiety is defined as an unpleasant state of uneasiness or tension that is secondary to a patient being concerned about a disease, hospitalization, anesthesia, and surgery, or the unknown.
Slor 2013 [29]	Y	Y	U	Y	NA	Y	U	Y	U	U	NA	Not mentioned
Van Grootven 2016 [32]	Y	Y	Y	Y	Y	Y	Y	Y	Y	Y	Y	Preoperative state anxiety reflects a temporary, acute anxious reaction with feelings of tension and apprehension.
Wada 2019 [10]	Y	Y	Y	Y	Y	U	Y	Y	Y	Y	Y	Not mentioned
Ackenbom 2022 [39]	Y	Y	Y	Y	Y	U	Y	U	Y	Y	Y	Not mentioned
Segernäs 2022 [40]	Y	Y	U	Y	Y	U	U	Y	U	U	Y	Not mentioned
Fukunaga 2022 [41]	Y	Y	Y	Y	Y	U	Y	N	Y	Y	Y	Preoperative anxiety is a common stress reaction to impending surgery

Q1: Were the two groups similar and recruited from the same population?

Q2: Were the exposures measured similarly to assign people to both exposed and unexposed groups?

Q3: Was the exposure measured in a valid and reliable way?

Q4: Were confounding factors identified?

Q5: Were strategies to deal with confounding factors stated?

Q6: Were the groups/participants free of the outcome at the start of the study (or at the moment of exposure)?

Q7: Were the outcomes measured in a valid and reliable way?

Q8: Was the follow up time reported and sufficient to be long enough for outcomes to occur?

Q9: Was follow up complete, and if not, were the reasons to loss to follow up described and explored?

Q10: Were strategies to address incomplete follow up utilized?

Q11: Was appropriate statistical analysis used?

*Y* Yes, *U* Unclear, *N* No, *NA* not applicable

### Assessments and incidences of preoperative anxiety and POD

The details of preoperative anxiety and POD assessment are presented in Table 1. Six tools were used to assess preoperative anxiety, including HADS-A (Anxiety subscale of Hospital Anxiety and Depression Scale, *n *= 6) [10, 11, 29, 37, 38, 40], STAI-S (State scale of Spielberger State-Trait Anxiety Inventory, *n* = 2) [12, 41], 6-item version of state scale of STAI (STAI-6, *n* = 1) [32], APAIS-A (Anxiety subscale of Amsterdam Preoperative Anxiety and Information Scale, *n* = 1) [13], VAS-A (Visual Analogue Scale for anxiety, *n* = 1) [13], and BAI (Beck Anxiety Inventory, *n* = 1) [39]. For the studies that reported the proportions of anxious patients, preoperative anxiety was defined as a HADS-A score of eight and greater on a maximum total score of 21 [10, 11, 38], STAI-S score of seven and greater on a maximum total score of 10 [12], and APAIS-A score of 11 and greater on a maximum total score of 20 [13]. Nine studies also reported the mean anxiety scores [10, 12, 13, 29, 32, 37, 39–41]. Five studies failed to report the explicit assessment time of preoperative anxiety [11, 37–40]. After contacting the authors, two of them responded and confirmed that preoperative anxiety had been assessed one day before surgery [37, 38]. The other studies reported the assessment time of preoperative anxiety to be the day before surgery or on average of 47.4 days (standard deviation = 30.5) before surgery for elective surgery [10, 12, 13, 41], within 12-24 h after admission but before surgery for emergency surgery [29, 32]. The incidence rates of preoperative anxiety ranged from 15 to 56% [10–13, 38].

Regarding the assessment of POD, a total of six instruments were used, of which the most used instruments were the CAM (*n* = 5) [11, 12, 29, 32, 39] and its adapted versions including CAM-ICU (CAM for the Intensive Care Unit, *n* = 6) [11–13, 37, 38, 40], and 3D-CAM (3-Minute Diagnostic Interview for CAM delirium, *n* = 1) [13]. Assessment time of POD varied from two to eight postoperative days, with a major focus on the first five days after surgery. The incidence of POD ranged from 7 to 43% [10–13, 29, 32, 37–41]. Seven studies reported duration of POD ranging from 2.0 to 3.3 days [10–12, 29, 32, 37, 38], and two studies reported the severity of POD [10, 32] (Table 1).

### Association between preoperative anxiety and POD

All studies used univariate analyses to investigate the difference between the delirium group and non-delirium group in terms of preoperative anxiety (Additional file 2). Ten of the studies showed no statistically significant differences between the two groups [10–13, 29, 32, 37, 39–41], while one study had the opposite result (*p* = 0.023) [38]. In addition, multivariable logistic regressions were performed in seven studies, and significant associations between preoperative anxiety and POD were reported in three studies [10, 11, 38], with preoperative anxiety being entered into a regression model as a dichotomous variable. Simultaneously no significant associations were reported in the other four [12, 13, 32, 41], and the adjusted ORs could be extracted from three of these studies [13, 32, 41] with preoperative anxiety being entered into a regression model as a continuous variable. More details on variables for which studies were adjusted and the reanalyzed results of included studies [12, 13, 32] in a different measurement level are also listed in Additional file 3.

### Meta-analysis

#### Meta-analysis for studies using dichotomized measurements of preoperative anxiety in multivariable analysis

**Fig. 2:**
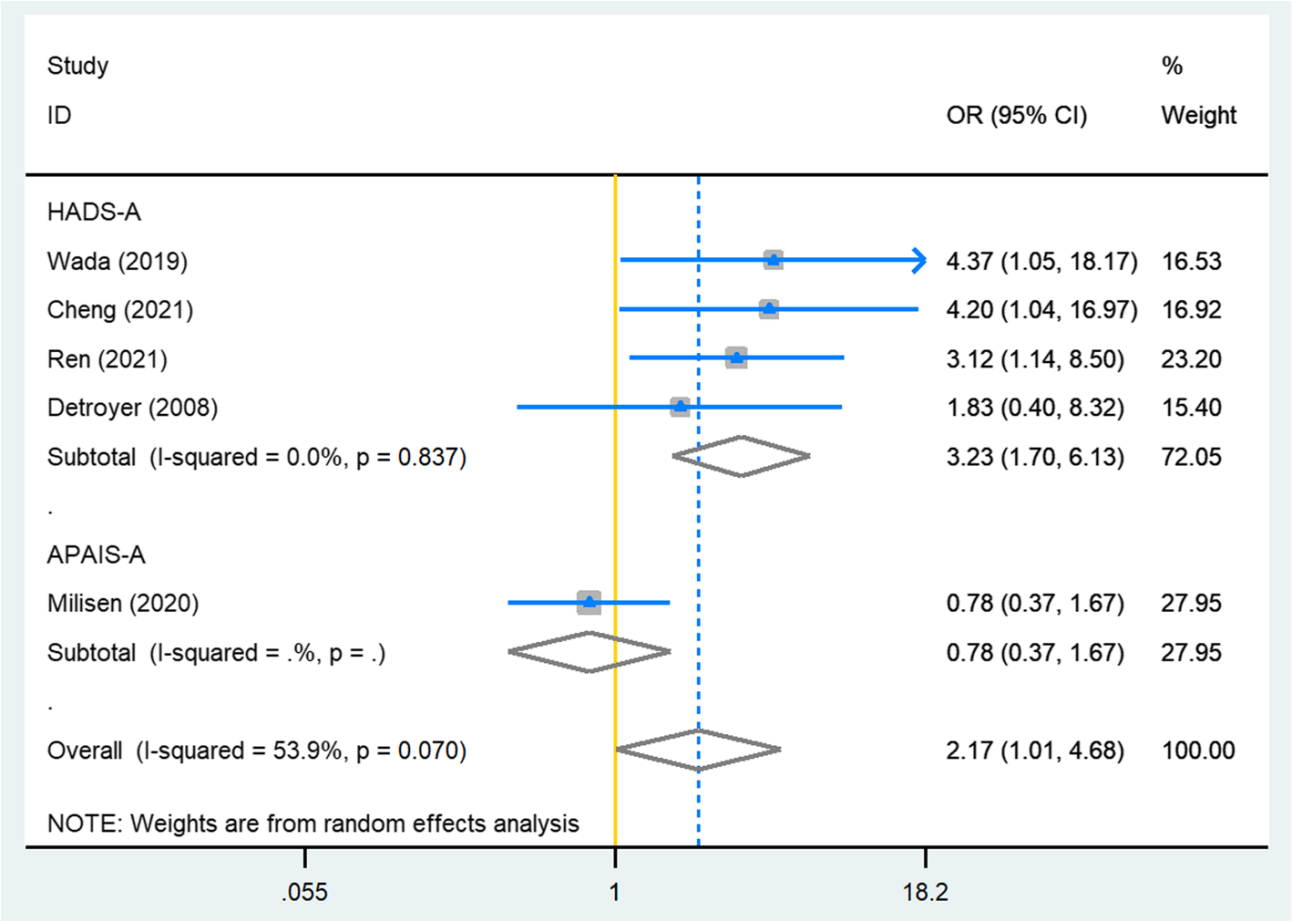
Meta-analysis for studies using dichotomized measurements of preoperative anxiety in multivariable analysis. HADS-A, Anxiety subscale of Hospital Anxiety and Depression Scale; APAIS-A, Anxiety subscale of Amsterdam Preoperative Anxiety and Information Scale; OR, odds ratio; CI, confidence interval; the recalculation of dichotomized measurements of HADS-A for Detroyer 2008 and APAIS-A for Milisen 2020 were conducted using multivariate logistic regression analysis adjusting for the same confounders as in the original analysis

Studies using dichotomous measurements of preoperative anxiety showed a significant positive association between preoperative anxiety and POD (Fig. 2, OR = 2.17, 95%CI: 1.01–4.68, *p* = 0.048, *n* = 5), although heterogeneity was at moderate level (I^2^ = 54%, Tau^2^ = 0.4). A significant association was also found when only studies were taken into account that used HADS-A (OR = 3.23, 95%CI: 1.70–6.13, *p* < 0.05, *n* = 4) without statistical heterogeneity (I^2^ = 0, Tau^2^ = 0).

Sensitivity analysis was restricted to the subgroup of HADS-A because the heterogeneity caused by different assessment tools may obscure other sources of heterogeneity. By removing the study of Ren 2021 [11] (failing to report the explicit preoperative assessment time), the major findings remained unchanged (Additional file 4).

#### Meta-analysis for studies using continuous measurements of preoperative anxiety in multivariable analysis

**Fig. 3:**
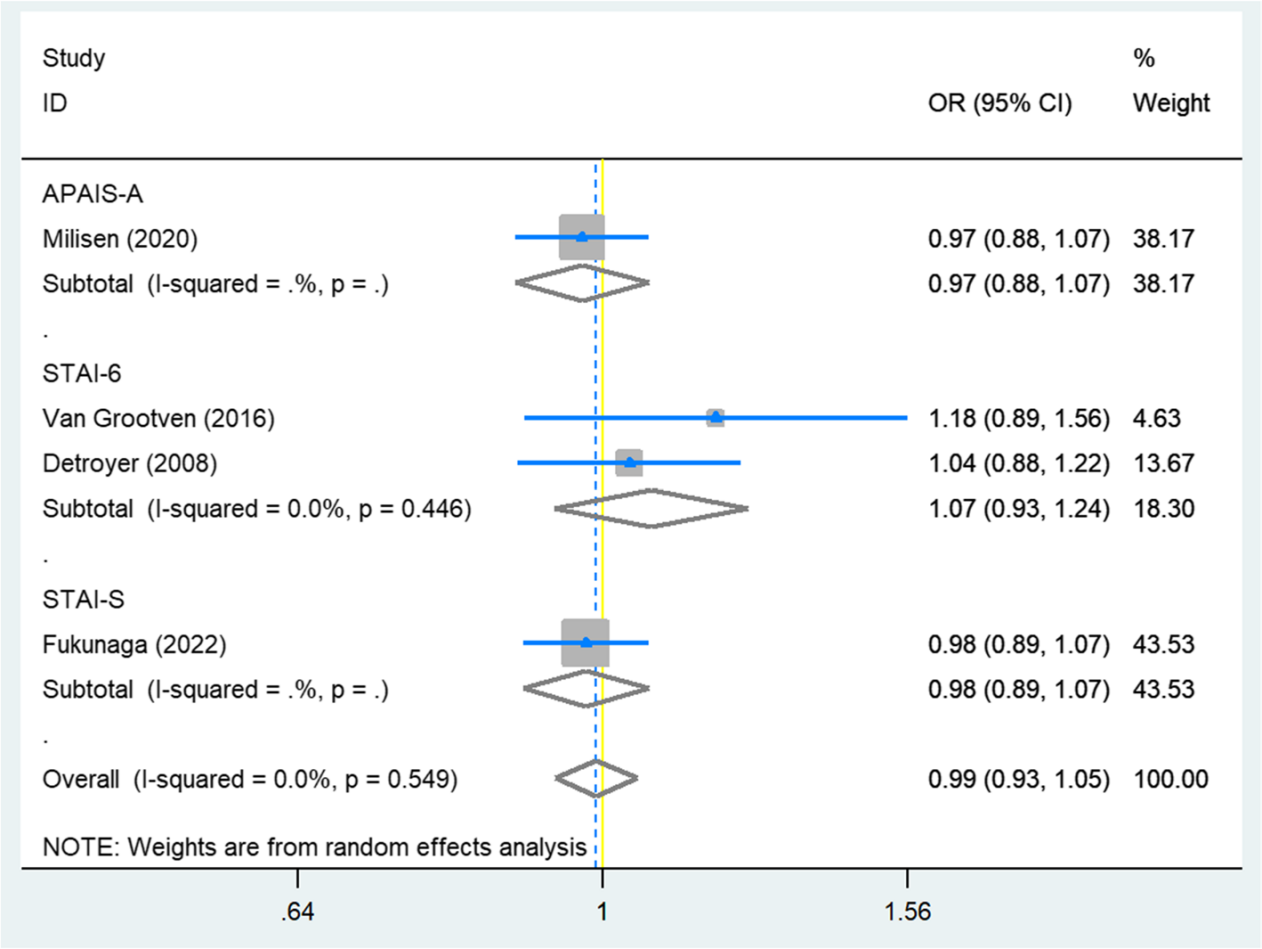
Meta-analysis for studies using continuous measurements of preoperative anxiety in multivariable analysis. APAIS-A, Anxiety subscale of Amsterdam Preoperative Anxiety and Information Scale; STAI-S, State scale of Spielberger State-Trait Anxiety Inventory; STAI-6, short form of state scale of STAI; OR, odds ratio; CI, confidence interval; we calculated STAI-6 instead of full version of STAI-S for Detroyer 2008 corresponding with the data from Van Grootven 2016 using multivariate logistic regression analysis adjusting for the same confounders as in the original analysis

We found no association between preoperative anxiety and POD according to the combined result of the studies using continuous predictors (Fig. 3; OR = 0.99, 95%CI: 0.93–1.05, I^2^ = 0, Tau^2^ = 0, *p* = 0.766, *n* = 4) and the subgroup analysis of studies using STAI-6 (OR = 1.07, 95%CI: 0.93–1.24, I^2^ = 0, Tau^2^ = 0, *p* = 0.323, *n* = 2). We conducted a sensitivity analysis by excluding Fukunaga 2022 [41] and the result was not reversed (Additional file 5; OR = 1.00, 95%CI: 0.93–1.09, I^2^ = 0, Tau^2^ = 0, *p* = 0.952, *n* = 3).

## Discussion

### Principal findings

The principal finding of our systematic review of 11 prospective studies and meta-analysis of seven studies was that there was an unclear association between preoperative anxiety and POD. While this association was significant only when dichotomous preoperative anxiety variables were utilized as measurements, it was not when continuous preoperative anxiety variables were used.

As a consequence, a definite conclusion on the relationship between preoperative anxiety and POD cannot be drawn and the following underlying mechanisms might warrant further research for this relationship. The neuroendocrine hypothesis, one of the main pathophysiological pathways of delirium, suggests that delirium represents a reaction to acute stress, mediated by abnormally high glucocorticoid levels, which may compromise the neuron's ability to survive various neurologic insults leading to or exacerbate cell death [28]. In addition, neuroinflammation triggered by peripheral inflammatory cytokines may over-activate the central nervous system leading to further neuronal injury, which is considered as an indirect mechanism for delirium [27, 28, 42]. Furthermore, evidence showed that anxiety could enhance the production of proinflammatory cytokines, including interleukin-6 which has been proven as a promising marker for delirium [26, 27, 43, 44]. What’s more, preoperative anxiety may cause sleep disturbance which has long been linked to the development of POD [28, 45, 46]. As such, the association between preoperative anxiety and POD is worthy of continued investigation.

### The definition and assessment tools of preoperative anxiety and POD

Unfortunately, the theoretical definitions of preoperative anxiety were only mentioned in five studies [11–13, 32, 41], while the others only referred in the manuscripts that patients’ anxiety was assessed before surgery using valid instruments without an explicit theoretical definition. In particular, preoperative anxiety was classified as “state anxiety”, as distinct from generalized anxiety or trait anxiety, in Detroyer 2008, Milisen 2020, and van Grootven 2016 [12, 13, 32]. The distinction between trait and state anxiety should gain prominence, as preoperative anxiety refers to state anxiety related to the condition of waiting to undergo anesthesia and surgery [47, 48]. Ambiguity in the conception of anxiety type may lead to inaccurate assessment of preoperative anxiety. State anxiety is referred to as a more transient intense emotional state encompassed feelings of tension, fear, and apprehension, along with a temporary heightened sympathetic nervous system activity; inversely, trait anxiety implies a generalized and enduring predisposition of nervous and anxiety as a personality feature [14, 49]. Trait anxiety is separate from state anxiety, but it is likely to contribute to state anxiety; ongoing research suggests that the interaction of these two types of anxiety is multidimensional, not straightforward, and several differences in the structural–functional patterns were found between them [49–51]. Hence, these two types of anxiety should not be conflated.

After defining and elucidating preoperative anxiety, the accuracy and appropriateness of the tools used to assess preoperative anxiety in the included studies needs further discussion. HADS, the most frequently used instrument across the included studies, is designed to screen for clinically significant anxiety and depression in non-psychiatric patients containing two subscales for anxiety and depression respectively [52]. STAI is widely used to measure anxiety related to either relatively stable personality characteristics or transitory emotional states triggered by stimuli, which succeed in separating state anxiety from trait anxiety [14]. BAI is developed for measuring the severity of anxiety including both physical and psychological symptoms of anxiety, with a focus on discriminating between anxiety and depression [53]. For the specific purpose of screening anxiety in the preoperative period, APAIS was developed, which contains both anxiety and the need for information components [54]. Comparing some items from these four scales (Additional file 6), it is evident that APAIS shows better construct validity with respect to the specific conditions confronted by surgical patients and is more patient-friendly and more targeted at preoperative anxiety compared to HADS-A, BAI and STAI-S [14, 52–54]. Items of HADS-A are related to generalized symptoms of anxiety, while items of STAI-S and BAI are about the presence and absence of anxiety at this moment, independent of specific triggers or context, which can be used to measure state anxiety under a wide range of changing stressful conditions [54–56]. Additionally, as indicated in the instructions of the scales, the HADS-A and BAI response is based on the patient's feelings during the past week [52, 53], while STAI-S and APAIS-A emphasize that the response should be based on the feelings at the time of assessment or during the preoperative period [14, 54]. However, in our study, we only find a significant association between preoperative anxiety and POD in studies using HADS-A, which may be due to the broader range of this anxiety measurement. The trade-off between a broad and/or narrow range of preoperative anxiety measure should be further discussed; and in future studies, it is imperative to choose the appropriate instrument to assess preoperative anxiety.

As for the assessment tool for POD, most of the included studies used the CAM and its adapted version, which has been acknowledged as the best bedside delirium assessment instrument [57, 58], such as CAM-ICU for critically ill patients [59] and 3D-CAM for ease of use [60]. The incidence rate of POD reported in the included studies corresponded to other studies, with the exception of one study in which the incidence rate was only 7% (10/152) in patients who had undergone cardiac surgery within five days after surgery [38, 61, 62]. A possible explanation might be that the mean age of participants in this study was younger than that of the cardiac surgical patients in other studies included in this review [12, 13, 37, 38]. However, the absence of definitive laboratory tests, the fluctuating course, and the broad differential diagnosis lead to only a fraction of patients with delirium that can be recognized [63, 64]. As a consequence, both the under-diagnosis of POD and preoperative anxiety make it more difficult to explore the relationship between them.

### Interpretation of results

The pooled results show an inconsistency between different levels of measurements (i.e. continuous scores vs. dichotomous scoring) used for preoperative anxiety assessment as a predictor of POD. Notably, despite a significant result being found between dichotomous anxiety measurements and POD, the reliability and interpretation of statistical data cannot be divorced from methodological limitations. Firstly, deciding on a cut-off may cause the loss of information and power to detect real relationships when converting continuous data to dichotomous data, so the statistical analysis of continuous variables is usually considered more powerful than the analysis of dichotomous variables [36, 65]. Further, the dichotomization of a continuous variable may increase the possibility of false-positive results and may lead to residual confounding in regression [65, 66]. Accordingly, reporting preoperative anxiety as a continuous variable may be more appropriate.

Additionally, there are some problems in selecting the appropriate variables from a list of candidate variables to enter the regression model. Including baseline variables that are considered clinically relevant based on expert clinical reasoning into the regression model may be the most preferable way [67, 68]. Cheng 2021 [38] failed to include preoperative cognitive functioning, one of the important confounders which has repeatedly been shown to be a risk factor of POD [69]. Fukunaga 2022 [41] reported that agreeableness was firstly detected to be involved in the development of POD as an independent psychological factor, suggesting that patients with lower agreeableness are predisposed to POD, which was not taken into account in the rest of included studies. On top of that, other covariates showing a univariate relationship with the outcome should also be entered into the regression model, but the threshold should be less stringent, such as *P* < 0.25, to avoid the neglect of important adjustment variables [68, 70]. Therefore, the strategies to select covariates in Chen 2021 [38] and Ren 2021 [11] might not be ideal, as both studies used *p* < 0.05 as a threshold to select covariates without considering the clinical relevance.

Apart from these methodological and statistical issues, three studies with significant differences in anxiety scores between delirious and non-delirious patients [10, 11, 38] were conducted in Asian countries, including two in China [11, 38] and one in Japan [10], while the other studies were conducted mostly in Europe and one in United States of America. Therefore, differences in culture or in the preparation before surgery might be another possible explanation for the differences found in the results of this review.

### Strengths and limitations

To our knowledge, this is the first systematic review and meta-analysis to investigate the association between preoperative anxiety and POD in older surgical patients. All the studies we combined were conducted using multivariable analysis. We registered our study protocol on PROSPERO before our study started, in order to avoid selective reporting bias.

Our results need to be interpreted with caution. First, there were differences in measurement levels for preoperative anxiety (i.e. continuous scores vs. dichotomous scoring), but we have done our utmost best to re-analyze data in order to reduce a source of heterogeneity and obtain a more valid pooled result. Second, our review may have another limitation due to conceptual differences among the preoperative anxiety measurement instruments regarding to the state and trait anxiety, which may cause uncertainty to the relationship between preoperative anxiety and POD. Third, although we combined all the adjusted ORs, which were considered more reliable results compared to crude ORs, different adjustments for potential confounders were conducted among the included studies, which may lead to bias for the pooled results. Forth, four studies didn’t include preoperative anxiety as a main predictor in their multivariable analyses because preoperative anxiety was not significant in their univariate analysis [29, 37, 39, 40], so we could not include these four studies in the meta-analysis.

## Conclusion

The results from our meta-analysis suggest that the association between preoperative anxiety and POD in older surgical patients is uncertain. Considering the ambiguity of the preoperative anxiety assessment instruments and the differences in results between dichotomous preoperative anxiety measurements and continuous measurements, further research is warranted in which a greater emphasis should be placed on how preoperative anxiety is operationalized and measured.

## Supplementary Information

**Additional file 1.** Search strategies for each database.

**Additional file 2.** The results of univariate analyses of included studies.

**Additional file 3.** The results of multivariable logistic regression of included studies.

**Additional file 4.** Sensitivity analysis of studies using dichotomous HADS-A for preoperative anxiety.

**Additional file 5.** Sensitivity analysis of studies using continuous predictors.

**Additional file 6.** Comparison of HADS, STAI, APAIS, and BAI.

## Data Availability

The datasets generated and/or analysed during the current study are available in the Zenodo repository,https://zenodo.org/record/7697317#.ZATwdGjMKUk.

## Abbreviations

POD: Postoperative delirium
PRISMA: Preferred Reporting Items for Systematic reviews and Meta-Analyses
CENTRAL: Cochrane Central Register of Controlled Trials
WHO-ICTRP: World Health Organization International Clinical Trials Registry Platform
JBI: Joanna Briggs Institute
OR: Odds ratios
CI: Confidence interval
STAI-S: State scale of Spielberger State-Trait Anxiety Inventory
STAI-6: 6-Item version of state scale of STAI
APAIS-A: Anxiety subscale of Amsterdam Preoperative Anxiety and Information Scale
VAS-A: Visual Analogue Scale for anxiety
HADS-A: Anxiety subscale of Hospital Anxiety and Depression Scale
BAI: Beck Anxiety Inventory
CAM: Confusion Assessment Method
CAM-ICU: CAM for the Intensive Care Unit
3D-CAM: 3-Minute Diagnostic Interview for CAM delirium
DSM-V: Diagnostic and Statistical Manual of Mental Disorders criteria fifth edition
