# Supplementary material for: Association between preoperative anxiety and postoperative delirium in older patients: a systematic review and meta-analysis
Source: BMC Geriatr. 2023 Mar 30;23:198. doi: 10.1186/s12877-023-03923-0 (PMC10064748; doi:10.1186/s12877-023-03923-0)
Supplement: Supplementary file 1 — Additional file 1. Search strategies for each database. [file 12877_2023_3923_MOESM1_ESM.docx]

Additional file 1 Search strategies for each database

| MEDLINE (via PubMed) | 1. "Anxiety"[Mesh] OR "Stress Disorders, Traumatic, Acute"[Mesh] OR "Panic"[Mesh] OR "Panic Disorder"[Mesh] 2. anxiet*[Title/Abstract] OR anxious*[Title/Abstract] OR panic*[Title/Abstract] OR stress*[Title/Abstract] OR distress*[Title/Abstract] OR fear*[Title/Abstract] OR nervous*[Title/Abstract] OR hypervigilance[Title/Abstract] OR astheni*[Title/Abstract] 3. #1 OR #2 4. "Delirium"[Mesh] OR "Cognitive Dysfunction"[Mesh] 5. delirium*[Title/Abstract] OR "acute confusion"[Title/Abstract] OR "acute organic psychosyndrome*"[Title/Abstract] OR "acute brain syndrome*"[Title/Abstract] OR "acute brain disease*"[Title/Abstract] OR "metabolic encephalopathy"[Title/Abstract] OR "acute psycho-organic syndrome*"[Title/Abstract] OR "clouded state"[Title/Abstract] OR "clouding of consciousness"[Title/Abstract] OR "exogenous psychosis"[Title/Abstract] OR "toxic psychosis"[Title/Abstract] OR "toxic confusion"[Title/Abstract] OR obnubilate*[Title/Abstract] OR delire[Title/Abstract] OR deliria[Title/Abstract] OR delirious[Title/Abstract] OR "emergence agitation"[Title/Abstract] OR "agitated emergence"[Title/Abstract] OR "emergence excitement"[Title/Abstract] OR "cognitive dysfunction*"[Title/Abstract] OR "cognitive impairment*"[Title/Abstract] OR "neurocognitive disorder*"[Title/Abstract] OR "cognitive decline*"[Title/Abstract] OR "mental deterioration*"[Title/Abstract] OR "cognitive deterioration*"[Title/Abstract] OR "cognitive defect*"[Title/Abstract] OR "cognition disorder*"[Title/Abstract] OR "cognitive disabilit*"[Title/Abstract] OR "cognitive disorder*"[Title/Abstract] 6. #4 OR #5 7. "Specialties, Surgical"[Mesh] OR "surgery" [Subheading] OR "Postoperative Care"[Mesh] OR "Postoperative Period"[Mesh] OR "Postoperative Complications"[Mesh] OR "Preoperative Care"[Mesh] OR "Preoperative Period"[Mesh] OR "Perioperative Period"[Mesh:NoExp] OR "Perioperative Care"[Mesh:NoExp] OR "Perioperative Nursing"[Mesh:NoExp] OR "Postanesthesia Nursing"[Mesh] OR "Perioperative Medicine"[Mesh] OR "Anesthesia"[Mesh] 8. surg*[Title/Abstract] OR operat*[Title/Abstract] OR postoperat*[Title/Abstract] OR postsurg*[Title/Abstract] OR "post procedur*"[Title/Abstract] OR postprocedur*[Title/Abstract] OR "after procedur*"[Title/Abstract] OR preoperat*[Title/Abstract] OR "pre procedur*"[Title/Abstract] OR preprocedur*[Title/Abstract] OR presurg*[Title/Abstract] OR "before procedur*"[Title/Abstract] OR perioperat*[Title/Abstract] OR "peri procedur*"[Title/Abstract] OR periprocedur*[Title/Abstract] OR perisurg*[Title/Abstract] OR narcos*[Title/Abstract] OR narcot*[Title/Abstract] OR anesthe*[Title/Abstract] OR anaesthe*[Title/Abstract] OR postanesthe*[Title/Abstract] OR postanaesthe*[Title/Abstract] 9. #7 OR #8 10. #3 AND #6 AND #9 |
| --- | --- |
| EMBASE (via Embase.com) | 1. 'anxiety'/de OR 'anticipatory anxiety'/exp OR 'anxiety disorder'/de OR 'acute stress disorder'/exp OR 'anxiety neurosis'/exp OR 'cardiac anxiety'/exp OR 'distress syndrome'/exp OR 'mixed anxiety and depression'/exp OR 'panic'/exp 2. anxiet*:ti,ab,kw OR anxious*:ti,ab,kw OR panic*:ti,ab,kw OR stress*:ti,ab,kw OR distress*:ti,ab,kw OR fear*:ti,ab,kw OR nervous*:ti,ab,kw OR hypervigilance:ti,ab,kw OR astheni*:ti,ab,kw 3. #1 OR #2 4. 'delirium'/de OR 'emergence agitation'/exp OR 'hyperactive delirium'/exp OR 'hypoactive delirium'/exp OR 'postoperative delirium'/exp OR 'cognitive defect'/de OR 'postoperative cognitive dysfunction'/exp 5. delirium*:ti,ab,kw OR 'acute confusion':ti,ab,kw OR 'acute organic psychosyndrome*':ti,ab,kw OR 'acute brain syndrome*':ti,ab,kw OR 'acute brain disease*':ti,ab,kw OR 'metabolic encephalopathy':ti,ab,kw OR 'acute psycho-organic syndrome*':ti,ab,kw OR 'clouded state':ti,ab,kw OR 'clouding of consciousness':ti,ab,kw OR 'exogenous psychosis':ti,ab,kw OR 'toxic psychosis':ti,ab,kw OR 'toxic confusion':ti,ab,kw OR obnubilate*:ti,ab,kw OR delire:ti,ab,kw OR deliria:ti,ab,kw OR delirious:ti,ab,kw OR 'emergence agitation':ti,ab,kw OR 'agitated emergence':ti,ab,kw OR "emergence excitement':ti,ab,kw OR 'cognitive dysfunction*':ti,ab,kw OR 'cognitive impairment*':ti,ab,kw OR 'neurocognitive disorder*':ti,ab,kw OR 'cognitive decline*':ti,ab,kw OR 'mental deterioration*':ti,ab,kw OR 'cognitive deterioration*':ti,ab,kw OR 'cognitive defect*':ti,ab,kw OR 'cognition disorder*':ti,ab,kw OR 'cognitive disabilit*':ti,ab,kw OR 'cognitive disorder*':ti,ab,kw 6. #4 OR #5 7. 'surgery'/exp OR 'postoperative complication'/exp OR 'anesthesia'/exp OR 'postanesthesia nursing'/exp OR 'postanesthesia care'/exp OR 'perioperative nursing'/exp OR 'perioperative medicine'/exp 8. surg*:ti,ab,kw OR operat*:ti,ab,kw OR postoperat*:ti,ab,kw OR postsurg*:ti,ab,kw OR 'post procedur*':ti,ab,kw OR postprocedur*:ti,ab,kw OR 'after procedur*':ti,ab,kw OR preoperat*:ti,ab,kw OR 'pre procedur*':ti,ab,kw OR preprocedur*:ti,ab,kw OR presurg*:ti,ab,kw OR 'before procedur*':ti,ab,kw OR perioperat*:ti,ab,kw OR 'peri procedur*':ti,ab,kw OR periprocedur*:ti,ab,kw OR perisurg*:ti,ab,kw OR narcos*:ti,ab,kw OR narcot*:ti,ab,kw OR anesthe*:ti,ab,kw OR anaesthe*:ti,ab,kw OR postanesthe*:ti,ab,kw OR postanaesthe*:ti,ab,kw 9. #7 OR #8 10. #3 AND #6 AND #9 11. #10 NOT 'conference abstract':it |
| Web of Science Core Collection (via Peking University) | Within editions: Science Citation Index Expanded (SCI-EXPANDED)—1900-present, Social Sciences Citation Index (SSCI)—1983-present, Arts & Humanities Citation Index (AHCI)—1983-present, Emerging Sources Citation Index (ESCI)—2015-present.   1. TS=("anxiet*" OR "anxious*" OR "panic*" OR "stress*" OR "distress*" OR "fear*" OR "nervous*" OR "hypervigilance" OR "astheni*") 2. TS=("delirium*" OR "acute confusion" OR "acute organic psychosyndrome*" OR "acute brain syndrome*" OR "acute brain disease*" OR "metabolic encephalopathy" OR "acute psycho-organic syndrome*" OR "clouded state" OR "clouding of consciousness" OR "exogenous psychosis" OR "toxic psychosis" OR "toxic confusion" OR "obnubilate*" OR "delire" OR "deliria" OR "delirious" OR "emergence agitation" OR "agitated emergence" OR "emergence excitement" OR "cognitive dysfunction*" OR "cognitive impairment*" OR "neurocognitive disorder*" OR "cognitive decline*" OR "mental deterioration*" OR "cognitive deterioration*" OR "cognitive defect*" OR "cognition disorder*" OR "cognitive disabilit*" OR "cognitive disorder*") 3. TS=("surg*" OR "operat*" OR "postoperat*" OR "postsurg*" OR "post procedur*" OR "postprocedur*" OR "after procedur*" OR "preoperat*" OR "pre procedur*" OR "preprocedur*" OR "presurg*" OR "before procedur*" OR "perioperat*" OR "peri procedur*" OR "periprocedur*" OR "perisurg*" OR "narcos*" OR "narcot*" OR "anesthe*" OR "anaesthe*" OR "postanesthe*" OR "postanaesthe*") 4. #1 AND #2 AND #3 |
| CINAHL Complete (via EBSCO) | 1. (MH "Anxiety") OR (MH "Anticipatory Anxiety") OR (MH "Anxiety Disorders") OR (MH "Panic Disorder") OR (MH "Stress+") 2. TI (anxiet* OR anxious* OR panic* OR stress* OR distress* OR fear* OR nervous* OR hypervigilance OR astheni*) OR AB (anxiet* OR anxious* OR panic* OR stress* OR distress* OR fear* OR nervous* OR hypervigilance OR astheni*) 3. #1 OR #2 4. (MH "Cognition Disorders") OR (MH "Delirium") 5. TI (delirium* OR "acute confusion" OR "acute organic psychosyndrome*" OR "acute brain syndrome*" OR "acute brain disease*" OR "metabolic encephalopathy" OR "acute psycho-organic syndrome*" OR "clouded state" OR "clouding of consciousness" OR "exogenous psychosis" OR "toxic psychosis" OR "toxic confusion" OR obnubilate* OR delire OR deliria OR delirious OR "emergence agitation" OR "agitated emergence" OR "emergence excitement" OR "cognitive dysfunction*" OR "cognitive impairment*" OR "neurocognitive disorder*" OR "cognitive decline*" OR "mental deterioration*" OR "cognitive deterioration*" OR "cognitive defect*" OR "cognition disorder*" OR "cognitive disabilit*" OR "cognitive disorder*") OR AB (delirium* OR "acute confusion" OR "acute organic psychosyndrome*" OR "acute brain syndrome*" OR "acute brain disease*" OR "metabolic encephalopathy" OR "acute psycho-organic syndrome*" OR "clouded state" OR "clouding of consciousness" OR "exogenous psychosis" OR "toxic psychosis" OR "toxic confusion" OR obnubilate* OR delire OR deliria OR delirious OR "emergence agitation" OR "agitated emergence" OR "emergence excitement" OR "cognitive dysfunction*" OR "cognitive impairment*" OR "neurocognitive disorder*" OR "cognitive decline*" OR "mental deterioration*" OR "cognitive deterioration*" OR "cognitive defect*" OR "cognition disorder*" OR "cognitive disabilit*" OR "cognitive disorder*") 6. #4 OR #5 7. (MH "Surgery, Operative+") OR (MH "Post Anesthesia Care") OR (MH "Postoperative Complications+") OR (MH "Perioperative Nursing") OR (MH "Perioperative Medicine") OR (MH "Anesthesia+") 8. TI (surg* OR operat* OR postoperat* OR postsurg* OR "post procedur*" OR postprocedur* OR "after procedur*" OR preoperat* OR "pre procedur*" OR preprocedur* OR presurg* OR "before procedur*" OR perioperat* OR "peri procedur*" OR periprocedur* OR perisurg* OR narcos* OR narcot* OR anesthe* OR anaesthe* OR postanesthe* OR postanaesthe*) OR AB (surg* OR operat* OR postoperat* OR postsurg* OR "post procedur*" OR postprocedur* OR "after procedur*" OR preoperat* OR "pre procedur*" OR preprocedur* OR presurg* OR "before procedur*" OR perioperat* OR "peri procedur*" OR periprocedur* OR perisurg* OR narcos* OR narcot* OR anesthe* OR anaesthe* OR postanesthe* OR postanaesthe*) 9. #7 OR #8 10. #3 AND #6 AND #9 |
| CENTRAL (via Cochrane Library) | 1. MeSH descriptor: [Anxiety] explode all trees OR MeSH descriptor: [Anxiety] explode all trees OR MeSH descriptor: [Panic] explode all trees OR MeSH descriptor: [Panic Disorder] explode all trees 2. (anxiet*):ti,ab,kw OR (anxious*):ti,ab,kw OR (panic*):ti,ab,kw OR (stress*):ti,ab,kw OR (distress*):ti,ab,kw OR (fear*):ti,ab,kw OR (nervous*):ti,ab,kw OR (hypervigilance):ti,ab,kw OR (astheni*):ti,ab,kw 3. #1 OR #2 4. MeSH descriptor: [Emergence Delirium] explode all trees OR MeSH descriptor: [Cognitive Dysfunction] explode all trees 5. (delirium*):ti,ab,kw OR ("acute confusion"):ti,ab,kw OR ("acute organic psychosyndrome*"):ti,ab,kw OR ("acute brain syndrome*"):ti,ab,kw OR ("acute brain disease*"):ti,ab,kw OR ("metabolic encephalopathy"):ti,ab,kw OR ("acute psycho-organic syndrome*"):ti,ab,kw OR ("clouded state"):ti,ab,kw OR ("clouding of consciousness"):ti,ab,kw OR ("exogenous psychosis"):ti,ab,kw OR ("toxic psychosis"):ti,ab,kw OR ("toxic confusion"):ti,ab,kw OR (obnubilate*):ti,ab,kw OR (delire):ti,ab,kw OR (deliria):ti,ab,kw OR (delirious):ti,ab,kw OR ("emergence agitation"):ti,ab,kw OR ("agitated emergence"):ti,ab,kw OR ("emergence excitement"):ti,ab,kw OR ("cognitive dysfunction*"):ti,ab,kw OR ("cognitive impairment*"):ti,ab,kw OR ("neurocognitive disorder*"):ti,ab,kw OR ("cognitive decline*"):ti,ab,kw OR ("mental deterioration*"):ti,ab,kw OR ("cognitive deterioration*"):ti,ab,kw OR ("cognitive defect*"):ti,ab,kw OR ("cognition disorder*"):ti,ab,kw OR ("cognitive disabilit*"):ti,ab,kw OR ("cognitive disorder*"):ti,ab,kw 6. #4 OR #5 7. MeSH descriptor: [Specialties, Surgical] explode all trees OR MeSH descriptor: [General Surgery] explode all trees OR MeSH descriptor: [Postoperative Care] explode all trees OR MeSH descriptor: [Postoperative Period] explode all trees OR MeSH descriptor: [Postoperative Complications] explode all trees OR MeSH descriptor: [Preoperative Care] explode all trees OR MeSH descriptor: [Preoperative Period] explode all trees OR MeSH descriptor: [Perioperative Period] this term only OR MeSH descriptor: [Perioperative Care] this term only OR MeSH descriptor: [Perioperative Nursing] this term only OR MeSH descriptor: [Perioperative Nursing] this term only OR MeSH descriptor: [Perioperative Medicine] explode all trees OR MeSH descriptor: [Anesthesia] explode all trees 8. (surg*):ti,ab,kw OR (operat*):ti,ab,kw OR (postoperat*):ti,ab,kw OR (postsurg*):ti,ab,kw OR ("post procedur*"):ti,ab,kw OR (postprocedur*):ti,ab,kw OR ("after procedur*"):ti,ab,kw OR (preoperat*):ti,ab,kw OR ("pre procedur*"):ti,ab,kw OR (preprocedur*):ti,ab,kw OR (presurg*):ti,ab,kw OR ("before procedur*"):ti,ab,kw OR (perioperat*):ti,ab,kw OR ("peri procedur*"):ti,ab,kw OR (periprocedur*):ti,ab,kw OR (perisurg*):ti,ab,kw OR (narcos*):ti,ab,kw OR (narcot*):ti,ab,kw OR (anesthe*):ti,ab,kw OR (anaesthe*):ti,ab,kw OR (postanesthe*):ti,ab,kw OR (postanaesthe*):ti,ab,kw 9. #7 OR #8 10. #3 AND #6 AND #9 |
