# Supplementary material for: Association between preoperative anxiety and postoperative delirium in older patients: a systematic review and meta-analysis
Source: BMC Geriatr. 2023 Mar 30;23:198. doi: 10.1186/s12877-023-03923-0 (PMC10064748; doi:10.1186/s12877-023-03923-0)
Supplement: Supplementary file 2 — Additional file 2. The results of univariate analyses of included studies. [file 12877_2023_3923_MOESM2_ESM.docx]

Additional file 2 The results of univariate analyses of included studies.

| Author year | Preoperative anxiety assessment | Non-delirium | Delirium | P-value/ OR (95% CI) |
| --- | --- | --- | --- | --- |
| Bakker 2012 | HADS-A score, mean (SD) | 6.9 (2.8) | 7.0 (2.3) | P=0.77 |
| Cheng 2021 | HADS-A＞7, n (%) | 24 (16.9) | 5 (50.0) | P=0.023 |
| Detroyer 2008 | STAI-S score, median (IQR) | 42 (17) | 38 (16) | P=0.52 |
| Milisen 2020 | APAIS-A score, mean (SD) | 8.9 (3.8) | 9.2 (3.8) | OR=0.98 (0.91–1.06) |
|  | APAIS-A score ≥ 11, n (%) | 21 (26.9) | 38 (33.9) | OR=0.72 (0.38–1.35) |
| Ren 2021 | HADS-A＞7, n (%) | 27 (14.2) | 13 (17.8) | P=0.467 |
| Slor 2013 | HADS-A score, mean (SD) | 9.8 (2.5) | 8.8 (1.7) | P=0.15 |
| Van Grootven 2016 | STAI-6 score, mean (SD) | 12.2 (2.0) | 12.5 (2.3) | OR=1.12 (0.89–1.41) |
| Wada 2019 | HADS-A, mean (SD) | 4.3 (3.0) | 5.3 (3.5) | P=0.158 |
|  | HADS-A＞7, n (%) | 7 (11.3) | 7 (24.1) | P=0.113 |
| Ackenbom 2022 | BAI score, median (range) | 4 (2-8.5) | 6.5 (3-12) | P=0.34 |
| Segernäs 2022 | HADS-A, median (range) | 4 (0–14) | 4 (0–14) | P=0.36 |
| Fukunaga 2022 | STAI-S score, mean (SD) | 37.0 (7.9) | 36.6 (8.3) | P=0.787 |

IQR, interquartile range; SD, standard deviation; OR, odds ratio; CI, confidence interval; STAI-S, State scale of Spielberger State-Trait Anxiety Inventory; STAI-6, 6-item version of state scale of STAI; APAIS-A, Anxiety subscale of Amsterdam Preoperative Anxiety and Information Scale; HADS-A, Anxiety subscale of Hospital Anxiety and Depression Scale; BAI, Beck Anxiety Inventory.
