# Supplementary material for: Association between preoperative anxiety and postoperative delirium in older patients: a systematic review and meta-analysis
Source: BMC Geriatr. 2023 Mar 30;23:198. doi: 10.1186/s12877-023-03923-0 (PMC10064748; doi:10.1186/s12877-023-03923-0)
Supplement: Supplementary file 3 — Additional file 3. The results of multivariable logistic regression of included studies. [file 12877_2023_3923_MOESM3_ESM.docx]

Additional file 3 The results of multivariable logistic regression of included studies.

| Author year | Assessment tool of exposure | Responses | Adjusted variables | Adjusted OR (95% CI) |
| --- | --- | --- | --- | --- |
| Bakker 2012 | HADS-A | mean score | not entered into multivariate analysis | - |
| Cheng 2021 | HADS-A | anxiety vs. no anxiety | EuroSCORE II, preoperative arrhythmia, length of ICU stay | 4.20 (1.04-16.97) |
| Detroyer 2008 | STAI-6 | mean score | living situation, time under cardiopulmonary bypass, smoking, alcohol, body temperature, glycemia, hemoglobin, APACHE II score at ICU admission, intubation time, and depressive symptoms | 1.04 (0.88-1.22)^*^ |
|  | HADS-A | anxiety vs. no anxiety |  | 1.83 (0.40-8.32)^*^ |
| Milisen 2020 | APAIS-A^#^ | mean score | age, preoperative cognitive functioning, depression preoperatively, premorbid activities of daily living, surgical risk, duration of cardiopulmonary bypass, diabetes mellitus and infection | 0.97 (0.88-1.07) |
|  |  | anxiety vs. no anxiety |  | 0.78 (0.35-1.60)^*^ |
| Ren 2021 | HADS-A | anxiety vs. no anxiety | age, BMI, ASA, CCI, education level, MMSE, preoperative anxiety, ICU admission, albumin, serum sodium, hemoglobin, blood transfusion, benzodiazepines, total fluid administration | 3.12 (1.14-8.50) |
| Slor 2013 | HADS-A | mean score | not entered into multivariate analysis | - |
| Van Grootven 2016 | STAI-6 | mean score | moderate level of education, MMSE, osteosynthesis surgery, lowest diastolic blood pressure, highest systolic blood pressure | 1.18 (0.89-1.56) |
| Wada 2019 | HADS-A | anxiety vs. no anxiety | age, MMSE, Duration of surgery, intraoperative DEX use, APACHE-II score, postoperative speech disability | 4.37 (1.05-18.18) |
| Ackenbom 2022 | BAI | mean score | not entered into multivariate analysis | - |
| Segernäs 2022 | HADS-A | mean score | not entered into multivariate analysis | - |
| Fukunaga 2022 | STAI-S | mean score | age; cerebrovascular disease; MMSE; STAI-Trait; agreeableness; APACHE-II score | 0.976 (0.891-1.070) |

STAI-6, 6-item version of state scale of Spielberger State-Trait Anxiety Inventory; APAIS-A, Anxiety subscale of Amsterdam Preoperative Anxiety and Information Scale; HADS-A, Anxiety subscale of Hospital Anxiety and Depression Scale; BAI, Beck Anxiety Inventory; STAI-S, State scale of Spielberger State-Trait Anxiety Inventory; APACHE II, Acute Physiology Age and Chronic Health Examination; MMSE, Mini Mental State Examination; ASA, American Society of Anesthesiology class; EuroSCPRE, European System for Cardiac Operative Risk Evaluation; BMI, Body Mass Index; CCI, Charlson Comorbidity Index; ICU, intensive care unit. OR, odds ratio; CI, confidence interval. *Reanalysis was conducted using multivariable logistic regression models with adjustments for the same potential confounders as in the original analysis; we calculated the 6-item version of STAI instead of full version of STAI-S for Detroyer 2008 so that it could correspond with the data from Van Grootven 2016, and we also calculated the adjusted OR of dichotomized measurement of HADS-A for Detroyer 2008 and dichotomized measurement of APAIS-A for Milisen 2020. ^#^Only the result of preoperative anxiety assessed by APAIS-A in Milisen 2020 was used.
