# Supplementary material for: Association between preoperative anxiety and postoperative delirium in older patients: a systematic review and meta-analysis
Source: BMC Geriatr. 2023 Mar 30;23:198. doi: 10.1186/s12877-023-03923-0 (PMC10064748; doi:10.1186/s12877-023-03923-0)
Supplement: Supplementary file 4 — Additional file 4. Sensitivity analysis of studies using dichotomous HADS-A for preoperative anxiety. [file 12877_2023_3923_MOESM4_ESM.docx]

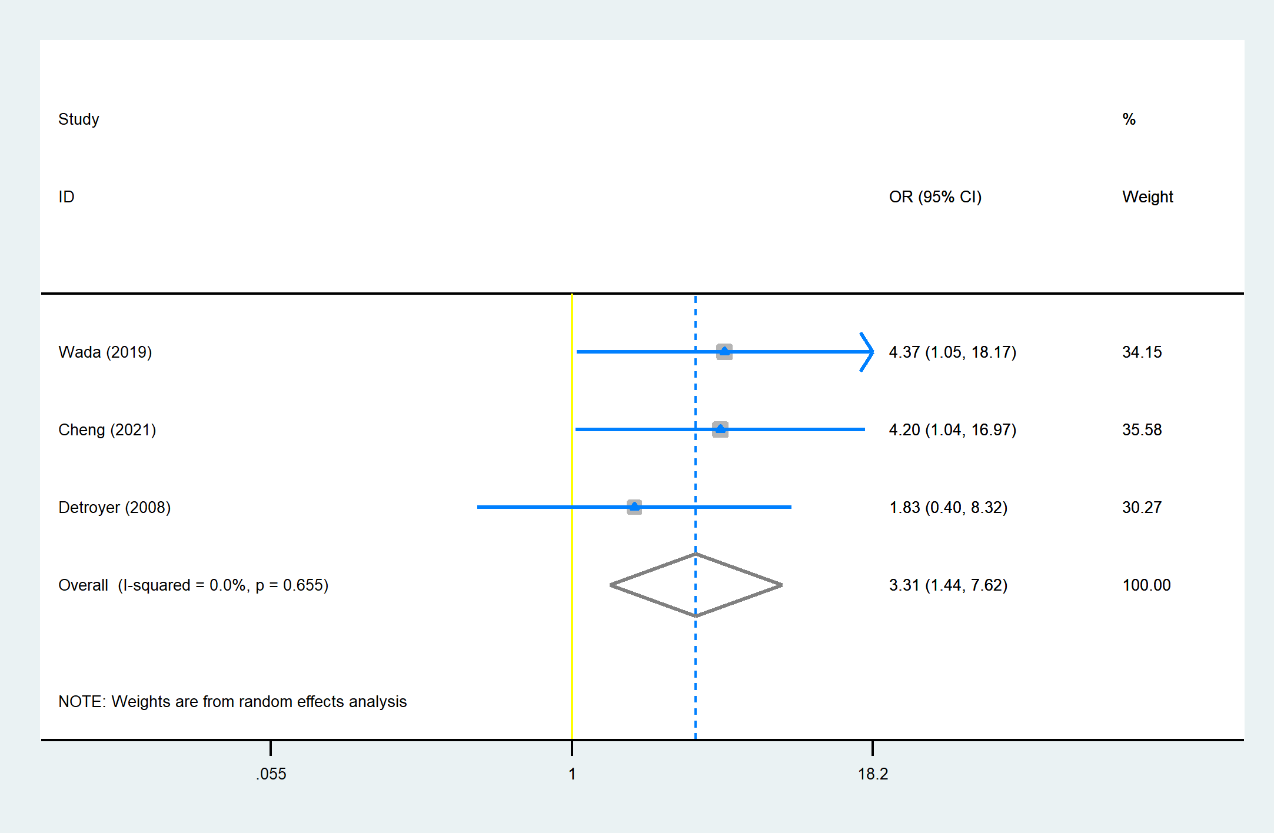


Additional file 4 Sensitivity analysis of studies using dichotomous HADS-A for preoperative anxiety.

OR, odds ratio; CI, confidence interval.
