# Supplementary material for: Association between preoperative anxiety and postoperative delirium in older patients: a systematic review and meta-analysis
Source: BMC Geriatr. 2023 Mar 30;23:198. doi: 10.1186/s12877-023-03923-0 (PMC10064748; doi:10.1186/s12877-023-03923-0)
Supplement: Supplementary file 6 — Additional file 6. Comparison of HADS, STAI, APAIS, and BAI. [file 12877_2023_3923_MOESM6_ESM.docx]

Additional file 6 Comparison of HADS, STAI, APAIS, and BAI.

|  | HADS-A | STAI-S | APAIS-A | BAI |
| --- | --- | --- | --- | --- |
| Number of items | 7 items | 20 items | 4 items | 21 items |
| Example of items | Worrying thoughts go through my mind. | I am worried. | I am worried about the anesthetic.  I am worried about the procedure. | Numbness or tingling |
|  | Do you ever feel tensed up or “wound up”? | I feel tense. | The anesthetic is on my mind continually.  The procedure is on my mind continually. | Unable to relax |
|  | Feel something awful is about happen? | I am presently worrying over possible misfortunes. |  | Feeling hot |
|  | Have panic attacks? | I feel frightened. |  | Fear of the worst happening |
| Response options | 0-not at all  1-from time to time, occasionally  2-a lot of the time  3-most of the time | 1-not at all  2-somewhat  3-moderately so  4-very much so | 1=not at all to 5=extremely | 0-not at all  1-mildly but it didn’t bother me much  2-moderately, it wasn’t pleasant at times  3-severely, it bothered me a lot |
| Response is based on | How you have been feeling in the past week. | How you feel right now, that is, at this moment. | How you feel in preoperative phase. | The physical and psychological symptoms over last week. |

STAI-S, State scale of Spielberger State-Trait Anxiety Inventory; APAIS-A, Anxiety subscale of Amsterdam Preoperative Anxiety and Information Scale; HADS-A, Anxiety subscale of Hospital Anxiety and Depression Scale; BAI, Beck Anxiety Inventory.
